# Physiological and behavioural effects of continuous remifentanil‐xylazine administration in donkeys

**DOI:** 10.1111/evj.70131

**Published:** 2025-11-27

**Authors:** Kássia Fernanda Araújo Damasceno, Andressa Nunes Mouta, Larissa de Sant' Ana Alves, Kathryn Nóbrega Arcoverde, Herbert Reis Aragão, Jerson Marques Cavalcante, Valéria Veras de Paula

**Affiliations:** ^1^ Department of Animal Science Universidade Federal Rural do Semi‐Árido (UFERSA) Mossoró Brazil

**Keywords:** asinine, horse, remifentanil, sedation, xylazine

## Abstract

**Background:**

Remifentanil and xylazine are used as continuous infusions to facilitate standing surgery in horses. Their use for this purpose has not been reported in donkeys.

**Objectives:**

To evaluate the behavioural, sedative, and cardiorespiratory effects of continuous intravenous infusion of remifentanil and xylazine in donkeys.

**Study Design:**

Non‐blinded in vivo experiments.

**Methods:**

Ten donkeys were sedated with an intravenous bolus of xylazine (0.8 mg/kg). After 3 min, continuous infusions of xylazine (0.65 mg/kg/h) and remifentanil (6 μg/kg/h) were administered for 60 min. Cardiorespiratory physiological parameters, rectal temperature, gastrointestinal motility, and sedation and ataxia scores were evaluated by a simple descriptive scale at M0 (baseline) and every 5 min, up to M60 (60 min), with scores 0–3. Head height concerning the ground was also evaluated. Dunnett and Friedman statistical tests (*p* < 0.05) were used.

**Results:**

Heart rate (*p* = 0.049) and respiratory rate (*p* = 0.001) decreased significantly at M10 and M5, respectively, compared to M0. There was a significant decrease in systolic (*p* = 0.04), mean (*p* = 0.02), and diastolic (*p* = 0.03) blood pressure at M15 compared to M0. The capillary refill time at M20 was statistically different (*p* = 0.001) from M0. The head height in relation to the ground reduced significantly from M5 (*p* = 0.001) to M60. Satisfactory sedation was obtained from M15 to M60. After stopping the infusion, all donkeys recovered successfully (7.1 ± 2.4 min). No adverse effects were observed during and after the infusion.

**Main Limitations:**

No painful stimulus or surgical procedure was performed.

**Conclusions:**

Combining remifentanil and xylazine at the doses used caused adequate sedation and short recovery time. Remifentanil did not cause excitation in the donkeys. Future studies are necessary to test the protocol with painful stimuli.

## 1 INTRODUCTION

Donkeys (*Equus asinus*) are rustic animals whose importance is often neglected due to their specific characteristics, both in morphophysiological and pharmacological perspectives, as well as their temperament.^1^ Thus, some studies have evaluated the effects of drugs on donkeys.^2^, ^3^ However, protocols for this species are still scarce.

It is common to administer drugs to chemically restrain equids, avoiding the complications and costs of general anaesthesia. The adrenergic alpha_2_‐agonist drugs produce sedation, analgesia, and muscle relaxation despite their adverse effects, such as bradycardia, hypotension and decreased gastrointestinal motility.^4^, ^5^, ^6^ Among adrenergic alpha_2_‐agonists, xylazine is the shortest acting and may result in insufficient analgesia for some surgical or outpatient procedures in monotherapy. In these circumstances, they are administered in association with opioids or with local blocking techniques to improve analgesia.^7^

Opioids are useful in anaesthesiology because of their ability to decrease sympathetic and somatic responses to harmful stimulation.^8^ Combinations of an alpha_2_‐agonist with an opioid in single or repeated administrations, or even as part of a peri‐anaesthetic protocol, can provide synergistic analgesic effects and good sedation.^6^ This association reduces the potential for excitation at the level of the central nervous system.^9^

Remifentanil is an opioid derivative of phenylpiperidine, supplied as remifentanil hydrochloride, a lyophilised powder. Animal studies indicate that the pharmacological properties of remifentanil are similar to those of other potent μ‐opioid receptor agonists, such as alfentanil.^10^ Characterised as a potent opioid of ultra‐short duration, its use has been described in studies conducted in different species, where it was shown to be a safe drug, producing minimal haemodynamic changes, in addition to its onset of action and rapid recovery after cessation of use, regardless of the duration of its administration. Therefore, remifentanil appears to be a highly titratable opioid, providing profound analgesia for very brief periods when needed for prolonged periods without the concern of delayed recovery.^8^, ^11^ Some studies describe its use in horses.^5^, ^6^, ^11^ However, the literature has no information on the systemic effects of remifentanil in donkeys.

This study aimed to evaluate the physiological and sedative effects of continuous intravenous infusion of remifentanil and xylazine in donkeys from the northeastern region of Brazil. We hypothesized that adverse effects would be minimal when the two drugs were administered at low doses by continuous infusion, and that this sedation protocol could be suitable for standing surgical procedures.

## 2 MATERIALS AND METHODS

### 2.1 Animals

Ten adult donkeys from the northeastern region of Brazil, seven males and three females, castrated, aged 5.3 ± 2.2 years and weighing 120.4 ± 21.4 kg, were used. The animals were classified as healthy after clinical and laboratory examinations (blood count, urea, creatinine, aspartate aminotransferase, alanine aminotransferase, and total proteins) for the inclusion criteria. A sample size calculation was performed based on a pilot study with three animals using previously published equine doses.^6^ The sample size calculation was performed using G*Power 3.1.9.7. The analysis was based on the mean and standard deviation of the sedation scores at 5 min after drug administration (M5), with an alpha level of 0.05 and a power of 0.8. Baseline values (M0), assumed to correspond to zero sedation, were used as the reference for comparison. The calculation indicated that a sample size of 10 animals was required. The animals were dewormed with ivermectin (Ivomec®, Boehringer Ingelheim), given trichlorfon and mebendazole (Trichlorsil paste®, Vansil), and vaccinated against rabies 4 weeks prior to the start of the study. The animals were housed in groups of five, in pickets of 10 × 7 m in the open air, with shade, and fed with voluminous grass (*Pennisetum purpureum*) and concentrate (ground corn, soybean meal, wheat bran, common salt, and calcitic limestone) twice a day, with water ad libitum. The acclimatisation period was 4 weeks.

### 2.2 Experimental study

One day before the procedure, the animals were transferred to individual stalls. Prior to the experiment, the animals were withheld from solid food for 12 h and had free access to water. At the beginning of the experimental day, the animal to be sedated was transferred to an adjacent room, where a restraint stock was located, and we waited an hour for the animal to acclimatise.

Initially, the physiological parameters of respiratory rate (RR) by inspection of the costal grid, capillary refill time (CRT), heart rate (HR) by cardiac auscultation, rectal temperature (RT), mean arterial pressure (MAP), systolic blood pressure (SBP), diastolic blood pressure (DBP) through a multiparametric monitor (multiparametric monitor, touch screen, Delta Life, model DL1000) were evaluated. Blood pressure was measured using the oscillometric method of the multiparameter monitor, with cuff number 5 positioned on the animal's tail. Intestinal sounds in the dorsal and ventral quadrants of the right and left sides were evaluated at baseline (M0), by the same evaluator in all animals and graded as absent, normal, decreased or increased. After M0, a 16‐gauge catheter coupled to a three‐way stopcock was aseptically inserted into the jugular vein.

Subsequently, the animals received a 0.8 mg·kg^−1^ *bolus* of xylazine (xylazine hydrochloride 2%, Syntec®) administered within 60 s by infusion pump (RS700 VET, RZVET®) and, after 3 min, continuous infusions of xylazine and remifentanil (remifentanil hydrochloride, Eurofarma®) were initiated at a rate of 0.65 mg/kg/h and 6 μg/kg/h, respectively, and discontinued over 60 min after 3 min of the initial xylazine bolus. The same physiological parameters evaluated at M0 (HR, RR, CRT, RT, MAP, SBP, and DBP) were monitored every 5 min until 60 min after drug administration (M60). Gastrointestinal motility was evaluated by auscultation at 15 min, 1, 4, 8, and 24 h after the infusions (M60). Head height above the ground was measured using a tape measure graduated in centimetres from 0 to 160 cm, which was fixed to the lateral bar of the physical containment stock to evaluate sedation. The distance between the ground and the mandibular symphysis was measured before beginning the instrumentation, in an environment free of external stimuli, considering the head height of the animal at M0. Sedation was also evaluated using a simple descriptive scale, assessing the level of sedation and ataxia,^5^, ^12^ with scores from 0 to 3 for both items (Table 1).

**TABLE 1:** Simple descriptive scale (SDS) scoring system for sedation and ataxia.^5^

	Signs
Sedation score	
0	No sedation. The animal is alert, mantains normal posture and responds appropriately to handling, normal objection to intervention.
1	Light sedation. Head lowered, facial muscles relaxed, and lower lip hanging. Some response to intervention.
2	Moderate sedation. Head lowered towards the floor, and swinging the hind legs. Slight response to intervention.
3	Marked sedation. Tries or becomes recumbent. No response to intervention.
Ataxia score	
0	No ataxia. The animal stands and walks normally; can rotate with force.
1	Mild ataxia. The animal can walk, but with some lack of limb control.
2	Moderate ataxia. The animal can only walk with support, staggers, but avoids falling.
3	Marked ataxia. The animal cannot walk without danger of falling, staggers, falls, turns over.

Recovery was evaluated from the end of the infusions to the time the patient returned to a score of zero for sedation and minimal ataxia. The sedation and ataxia scores were assessed every 2 min after stopping the continuous infusion of xylazine and remifentanil. Any need for more drugs for sedation, decreased administration, or any unexpected effect was also reported from the first xylazine *bolus* until recovery.

### 2.3 Data analysis

Data were expressed as mean ± standard deviation, median, minimum, and maximum using the SAS statistical software v9 (System for Windows, SAS Institute). After verifying the assumptions of parametric analysis, each variable was evaluated for statistical time points (M0–M60). Parametric data, were analysed using a linear mixed‐effects model for repeated measures (Proc Mixed procedure of the SAS program) followed by Tukey's test. Nonparametric data were analysed using the Friedman test. The level of significance was set at 5%.

## 3 RESULTS

Sedation and ataxia were satisfactory in all animals throughout the procedure, starting soon after xylazine administration. From 20 min onward, both parameters reached a median score of 2, which was maintained until the end of the infusion (Table 2).

**TABLE 2:** Mean ± standard deviation (SD), median, minimum and maximum (min–max) for sedation score and ataxia score of donkeys submitted to continuous infusion of remifentanil and xylazine for 60 min.

Variables	Measures	M0	M5	M10	M15	M20	M30	M45	M60
Sedation score	Mean	0	0.9	1.0	1.2	1.6	1.7	1.9	1.9
(0–3)	±SD	±0^b^	±0.6^a^	±0.5^a^	±0.4^a^	±0.5^a^	±0.5^a^	±0.3^a^	±0.3^a^
	Median	0	1	1	1	2	2	2	2
	Min–max	0–0	0–2	0–2	1–2	1–2	1–2	1–2	1–2
Ataxia score	Mean	0	0.8	1.0	1.1	1.6	1.7	1.8	1.9
(0–3)	±SD	±0^c^	±0.6^b^	±0.5^ab^	±0.6^ab^	±0.5^ab^	±0.5^ab^	±0.4^ab^	±0.3^a^
	Median	0	1	1	1	2	2	2	2
	Min–max	0–0	0–2	0–2	0–2	1–2	1–2	1–2	1–2

*Note*: Different lowercase letters in the line mean statistical difference (*p* < 0.05—Friedman).

Heart rate decreased significantly (*p* = 0.049) at M10 and remained lower than baseline values until M60 (Table 3). Respiratory rate showed an early and marked reduction, with a significant decrease at M5 (*p* < 0.001) that persisted until the end of the observation period (Table 3). Systolic, mean, and diastolic arterial pressures also decreased significantly from M15 onward (SBP: *p* = 0.04; MAP: *p* = 0.02; DBP: *p* = 0.03) and remained lower until M60, with the exception of MAP at M30, where no statistical difference was detected; however, the reduction returned at subsequent time points (Table 3).

**TABLE 3:** Mean values ± standard deviation (SD), median, minimum and maximum (min–max) for heart rate (HR), respiratory rate (RR), systolic blood pressure (SBP), mean arterial pressure (MAP) and diastolic pressure (DBP) of donkeys submitted to continuous infusion of remifentanil and xylazine in 60 min.

Variables	Measures	M0	M5	M10	M15	M20	M25	M30	M35	M40	M45	M50	M55	M60
HR (bpm)	Mean ± SD	44.4 ± 6.22^a^	40.9 ± 6.1^ab^	39.8 ± 7.87^b^	39.3 ± 7.06^b^	39.5 ± 8.92^b^	39.4 ± 9.02^b^	37.1 ± 8.37^b^	39.0 ± 8.55^b^	37.1 ± 9.41^b^	38 ± 7.83^b^	39.5 ± 8.58^b^	38.5 ± 9.63^b^	41.5 ± 13.63^b^
	Median	41.5	40	39	37.5	37	36.5	35.5	37	37	35.5	36	35.5	37
	Min–max	38–56	34–56	30–57	31–53	30–56	30–56	28–55	29–55	20–55	29–55	30–56	29–59	30–67
RR (rpm)	Mean ± SD	34.3 ± 7.6^a^	18.0 ± 9.0^b^	15.3 ± 7.83^bc^	14.1 ± 7.95^bc^	13.8 ± 6.48^bc^	12.0 ± 6.9^c^	11.0 ± 5.12^c^	11.0 ± 5.68^c^	10.6 ± 6.15^c^	9.8 ± 5.73^c^	9.6 ± 5.17^c^	10.4 ± 6.72^c^	10.5 ± 6.33^c^
	Median	35	14	11.5	11	10.5	9	8.5	9.5	9.5	8	7.5	7.5	8
	Min–max	25–48	9–32	7–28	6–28	8–25	5–27	7–20	5–23	4–25	5–24	5–22	6–27	6–27
SBP^a^ (mmHg)	Mean ± SD	166.8 ± 23.47^a^	160.7 ± 34.17^ab^	153.7 ± 28.53^abc^	137.6 ± 34.36^bc^	136.3 ± 28.35^bc^	144.7 ± 33.32^bc^	144.8 ± 32.79^bc^	132.2 ± 25.29^bc^	125.9 ± 32.76^c^	147.3 ± 39.91^bc^	142.1 ± 43.79^bc^	135.44 ± 33.14^bc^	131.7 ± 30.34^bc^
	Median	168.5	174	153.5	133.5	141.5	139.5	137	133	124.5	135	122	121	117.5
	Min–max	128–196	92–202	114–200	90–189	90–167	99–205	98–192	87–176	83–188	97–204	91–204	107–205	106–188
MAP^a^ (mmHg)	Mean ± SD	137.9 ± 32.01^a^	141.4 ± 41.76^a^	133.6 ± 31.28^a^	113.9 ± 38.11^b^	109.9 ± 32.42^b^	116.3 ± 47.02^b^	128.2 ± 37.3^b^	112.1 ± 30.97^b^	107.9 ± 38.88^b^	123.3 ± 35.86^b^	109.1 ± 36.49^b^	111.56 ± 34.16^b^	108.9 ± 33.82^b^
	Median	143.5	146.5	131	107	102	91	120	106	89	107	98.5	105	104
	Min–max	83–169	92–197	83–179	64–178	73–163	73–201	83–192	73–159	58–177	73–172	64–167	64–177	73–181
DBP (mmHg)	Mean ± SD	118.8 ± 40.98^a^	106.5 ± 40.12^a^	110.4 ± 29.7^a^	99.1 ± 37.23^b^	95.7 ± 30.51^b^	100.5 ± 48.5^b^	101 ± 39.4^b^	98.2 ± 31.24^b^	87.4 ± 37.86^b^	93.6 ± 26.97^b^	87.6 ± 32.27^b^	85.44 ± 35.75^b^	86.8 ± 27^b^
	Median	122	102	113.5	87.5	87.5	73.5	90.5	87.5	75	83	76.5	76	81
	Min–max	58–172	66–172	59–148	56–166	57–146	59–197	57–189	60–151	47–151	66–135	43–137	52–163	59–145

*Note*: Different lowercase letters in the line mean statistical difference (*p* < 0.05—Tukey).

Abbreviations: FC, bpm; FR, bpm.

^a^ Friedman.

Rectal temperature remained stable throughout the procedure (Table 4). The head height relative to the ground was significantly reduced compared to baseline (*p* < 0.001) and remained lower throughout the entire infusion period (Table 4). Animals recovered from sedation 7.1 ± 2.4 min after discontinuation of the infusion and were able to ambulate with minimal ataxia.

**TABLE 4:** Mean values ± standard deviation (SD), median, minimum and maximum (min – max) for capillary refill time (CRT), rectal temperature (RT) and head height of donkeys submitted to continuous infusion of remifentanil and xylazine in 60 min.

Variables	Measures	M0	M5	M10	M15	M20	M25	M30	M35	M40	M45	M50	M55	M60
CRT^a^ (s)	Mean ± SD	2.6 ± 0.7^a^	2.05 ± 0.37^ab^	1.95 ± 0.16^b^	2.05 ± 0.37^ab^	1.95 ± 0.16^b^	1.75 ± 0.42^b^	1.80 ± 0.59^b^	1.85 ± 0.53^b^	1.85 ± 0.24^b^	1.70 ± 0.35^b^	1.60 ± 0.39^b^	1.70 ± 0.35^b^	1.80 ± 0.26^b^
	Median	2.5	2	2	2	2	2	2	2	2	1.75	1.5	1.75	2
	Min–max	2–4	1.5–3	1.5–2	1.5–3	1.5–2	1–2	1–3	1–3	1.5–2	1–2	1–2	1–2	1.5–2
RT (°C)	Mean ± SD	36.98 ± 0.37^a^	37.03 ± 0.64^a^	36.89 ± 0.49^a^	36.96 ± 0.54^a^	36.91 ± 0.63^a^	36.67 ± 0.69^a^	36.59 ± 0.68^a^	36.5 ± 0.68^a^	36.56 ± 0.49^a^	36.61 ± 0.55^a^	36.31 ± 0.61^a^	36.31 ± 0.57^a^	36.19 ± 0.82^a^
	Median	37.1	36.9	36.85	37	36.75	36.7	36.55	36.4	36.6	36.75	36.2	36.05	36.05
	Min–max	36.5–37.4	35.9–37.9	36.2–37.8	36.2–38	36.1–38	35.8–37.6	35.7–37.8	35.4–37.5	35.8–37.1	35.8–37.3	35.6–37.1	35.6–37	34.7–37.2
Head height (cm)	Mean ± SD	83.0 ± 9.49^a^	28.7 ± 28.27^b^	28.78 ± 33.12^b^	18.11 ± 25.27^b^	16 ± 22.17^b^	15.56 ± 20.98^b^	17 ± 21.27^b^	17.5 ± 19.89^b^	16.67 ± 21.35^b^	16.56 ± 21.42^b^	16.44 ± 21.5^b^	16.22 ± 18.83^b^	14.44 ± 17.31^b^
	Median	82.5	15	15	5	5	5	10	10	5	5	5	8	10
	Min–max	65–100	0–85	0–84	0–78	0–69	0–65	0–63	0–63	0–63	0–63	0–63	0–56	0–56

*Note*: Different lowercase letters in the line mean statistical difference (*p* < 0.05—Tukey).

^a^ Friedman.

No adverse effects were observed during or after the procedure. Following recovery, behaviour, physiological parameters (HR, RR, SBP, DBP, MAP, and RT), appetite, and gastrointestinal sounds returned to baseline values and remained stable.

## 4 DISCUSSION

Use of alpha_2_‐agonists and opioids has previously been studied in donkeys; however, this is the first study to evaluate remifentanil in this species. The doses of xylazine and remifentanil were based on a previous study in horses. Objective assessment and quantification of sedation in donkeys are challenging, since no validated scoring system exists for this species. Nevertheless, our findings demonstrate that continuous infusion of xylazine and remifentanil has potential for sedating donkeys during standing procedures.^13^, ^14^ A moderate degree of sedation was observed in a preliminary study conducted with two donkeys, suggesting that higher doses of this association are unnecessary in this species.^6^ The results obtained were satisfactory regarding sedation and ataxia, thus corroborating work by Lizagarra and Castillo‐Alcala^13^ who also used xylazine, with an opioid, butorphanol, in donkeys. In a study evaluating the combination of butorphanol with dexmedetomidine or xylazine for standing unilateral ovariectomy in donkeys reported moderate to severe sedation (score 2–3).^14^ Both protocols have been proposed for standing procedures in donkeys.^14^ Although the opioid administered in that study^14^ differs from the one used in ours, the selected xylazine dose was lower (0.5 mg/kg bolus followed by 0.5 mg/kg/h) and during the continuous infusion, the rate was further decreased to 0.32 mg/kg/h. Similar results have been found when combining xylazine with different doses of butorphanol as boluses.^13^

In the current study, the depth of sedation was also objectively assessed using head height relative to the ground, as reported previously in horses.^15^ Although sedation appeared to be effective and additional doses of drugs were not necessary, no painful stimulus or surgical procedure was performed, which is an important limitation of the current study. Pallarols et al.^6^ used the same protocol in two horses undergoing standing laparoscopy; one of these animals required an additional dose of 0.25 mg/kg of xylazine.

There was little evidence of any serious cardiovascular or respiratory effects. Slowing the heart rate is a well‐known effect of adrenergic alpha‐2 agonists.^16^ However, heart rate decreased significantly from baseline only at 5 min, after which it remained stable throughout the infusion. This may be related to the doses selected and the slow administration of the initial bolus. Moreover, the heart rate reduction was not likely to have clinical repercussions and heart rate remained within the appropriate range for the species.

Opioid‐related adverse effects such as excitation, increased motor activity, or muzzle tremors were not observed in these donkeys, differing from previous studies^14^ with horses. Narcotic analgesics have been associated with excitation and unpredictable reactions in horses. Stimulation of the central nervous system and increased locomotor capacity are highly predictable and dose‐related responses after administration of most narcotic analgesics in horses.^17^ There is also some reluctance to use opioids in horses due to possible adverse effects including decreased gastrointestinal motility.^18^ During the current study, gastrointestinal motility was assessed by auscultation before and after the infusions. Gastrointestinal motility was also evaluated within 24 h after drug administration with no changes found in these animals.

Although sedation and ataxia increased progressively during the infusion, recovery after discontinuation of drug administration was rapid (7.1 ± 2.4 min). This is expected with remifentanil, which has an ultra‐short elimination half‐life due to rapid metabolism by non‐specific plasma and tissue esterases.^19^ In horses, remifentanil infusion is followed by rapid recovery regardless of infusion duration,^20^, ^21^ aligning with our findings in donkeys. Xylazine likely contributed to the progressive sedation and ataxia; however, its effects also subside quickly once administration is discontinued.^22^, ^23^

Previous work has demonstrated that donkeys may exhibit pharmacodynamic responses to α2‐agonists distinct from those observed in horses, which further justifies the need for species‐specific studies.^24^ Taken together, these findings highlight the unique pharmacological profile of the xylazine–remifentanil combination and emphasise the importance of further pharmacokinetic and pharmacodynamic investigations in donkeys. Our data also parallel a previous study^7^ in which blood concentrations of remifentanil during and after infusion in horses anaesthetised with isoflurane and dexmedetomidine decreased rapidly after the infusion, consistent with its short half‐life (12.8 ± 2.1 min). However, pharmacokinetic studies have not yet been performed in donkeys.

## 5 CONCLUSION

The association of xylazine and remifentanil at the doses used caused adequate sedation in donkeys from the northeastern region of Brazil during standing procedures. Remifentanil did not cause excitation in donkeys. Further studies are needed to assess this protocol in donkeys experiencing painful stimuli.

## FUNDING INFORMATION

The Article Processing Charge for the publication of this research was funded by the Coordenação de Aperfeiçoamento de Pessoal de Nível Superior ‐ Brasil (CAPES) (ROR identifier: 00x0ma614).

## CONFLICT OF INTEREST STATEMENT

The authors declare no conflicts of interest.

## AUTHOR CONTRIBUTIONS

**Kássia Fernanda Araújo Damasceno:** Writing – original draft; data curation; investigation; methodology. **Andressa Nunes Mouta:** Writing – original draft; data curation; investigation. **Larissa de Sant' Ana Alves:** Data curation; investigation. **Kathryn Nóbrega Arcoverde:** Investigation; writing – original draft; data curation. **Herbert Reis Aragão:** Data curation; investigation. **Jerson Marques Cavalcante:** Data curation; investigation. **Valéria Veras de Paula:** Conceptualization; writing – review and editing; methodology.

## DATA INTEGRITY STATEMENT

Valéria Veras de Paula had full access to all the data in the study and takes responsibility for the integrity of the data and the accuracy of data analysis.

## ETHICAL ANIMAL RESEARCH

The study was approved by the Animal Use Ethics Committee (CEUA/UFERSA) under protocol number 42/22.

## INFORMED CONSENT

Not applicable.

## Data Availability

The data that support the findings of this study are openly available in Zenodo following acceptance at https://doi.org/10.5281/zenodo.17693763.
